# Case report: Mycetoma caused by *Gordonia soli*

**DOI:** 10.1590/0037-8682-0326-2023

**Published:** 2023-09-22

**Authors:** João César Beenke França, Bruno Hassunuma Carneiro, Regielly Caroline Raimundo Cognialli, Flávio de Queiroz-Telles

**Affiliations:** 1 Universidade Federal do Paraná, Hospital de Clínicas, Curitiba, PR, Brasil.; 2 Universidade Federal do Paraná, Departamento de Saúde Coletiva, Curitiba, PR, Brasil.

**Keywords:** Mycetoma, Gordonia soli, Actinomycetoma

## Abstract

Mycetoma is a neglected tropical disease caused by fungi (eumycetoma) or bacteria (actinomycetoma), with high morbidity. *Gordonia* spp. are gram-positive bacteria that have previously been reported to cause mycetoma. Here, we report a case of *Gordonia soli* (initially misidentified as *Nocardia* spp.) as the etiological agent of actinomycetoma in a 64-year-old patient. After a literature search in the Cochrane Library, LILACS, SciELO, MEDLINE, PubMed, and PubMed Central databases, we concluded that this is the first case report of mycetoma caused by *Gordonia soli*. The current case highlights the importance of microbiological diagnosis of mycetoma and the challenges in its management.

## INTRODUCTION

Mycetoma is a neglected implantation tropical disease characterized by a chronic, granulomatous, tissular response. The route of infections is usually the transcutaneous inoculation of sapronotic organisms, including several species of bacteria and fungi. The etiologic agents of mycetoma usually are traumatically inplanted trhough splinters, thorns an plant debris.The most commonly involved anatomical regions are the legs and feet, although the shoulders and upper back may also be affected when carrying wood logs or lying down in direct contact with the soil in endemic regions^1^. The lesion progresses over months or even years after the traumatic injury, usually evolving from painless enlarging nodules to purulent lesions with draining fistulas and elimination of grains of variable sizes. It can be caused by fungi (Eumycetoma) or bacteria (Actinomycetoma)^2^ ^,^ ^3^.

Most mycetoma cases have been reported in Mexico, India, and Sudan^1^ ^,^ ^3^. In Mexico and India, 97% and 58% of the cases, respectively, were actinomycetomas, whereas in Sudan, 73% were eumycetoma^3^. In Brazil, nearly 300 mycetoma cases have been reported, with a high prevalence of actinomycetoma (75%)^4^.

The presumptive diagnosis of the microbiologic etiology of both actinomycetoma and eumycetoma can be made based on the morphological features of direct grain microscopic examination^5^. The causative agent of the mycetoma was identified through isolation from biological specimens^5^. Genus and species identification, which was historically performed using biochemical tests, can be achieved by molecular methods such as16S rRNA or Internal Transcribed Spacer (ITS) gene sequencing or matrix-assisted laser desorption ionization-time of flight mass spectrometry (MALDI-TOF MS)^5^ ^,^ ^6^. Actinomycetoma is most commonly caused by *Nocardia brasiliensis, Streptomyces somaliensis* and *Actinomadura madurae* ^1^ ^,^ ^7^.

Here, we present a case of mycetoma caused by *Gordonia soli*, initially identified as *Nocardia* spp*.* Additionally, a literature review was performed using the Cochrane Library, LILACS, SciELO, MEDLINE, PubMed, and PMC (PubMed Central) databases.

## CASE REPORT

**FIGURE 1:**
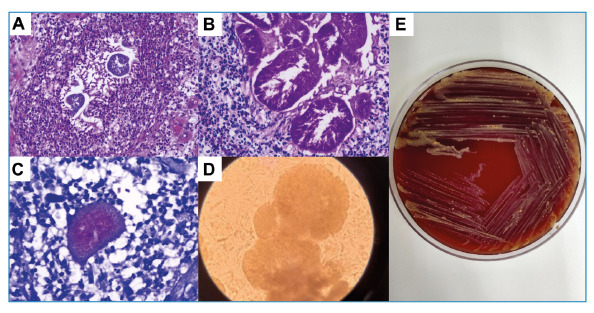
Microbiological and histopathological findings; **(A)** Grain in hematoxylin and eosin stain; **(B)** Grain in periodic Acid-Schiff stain; **(C)** Grain in Fite-Faraco stain; **(D)** Direct microcopy with 40% potassium hydroxide showing white homogenous grain; **(E)** Colonies of *Gordonia soli* in blood agar medium culture after 72 h of incubation at 30ºC.

A 64-year-old man with no comorbidities presented with painful nodular lesions on his left foot following a traumatic injury. On examination, there were enlarging nodules that progressed to purulent lesions with sanguinolent discharge, draining fistulas, and elimination of grains. A clinical diagnosis of mycetoma was made and a biopsy was performed. The histopathological evaluation revealed granulomas and fibrosis. It was also remarkable for the presence of grains and filamentous structures compatible with an actinomycetoma (Figure 1-A, B,C). Multiple tissue cultures were negative, and the causative agent was presumed to be *Nocardia* spp*.* based on clinical and histopathological findings.

**FIGURE 2:**
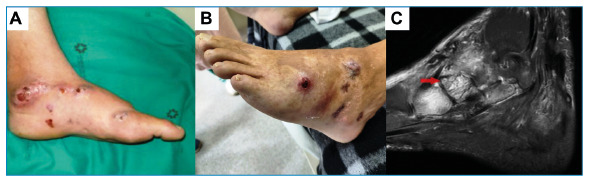
Clinical features; **(A)** Nodular lesions on left foot compatible with mycetoma; (B) Recurrence of symptoms after several years of medical treatment; (C) Magnetic resonance imaging (MRI) of left foot, revealing high-signal areas on fat-saturated T2 weighted sequence in the subcutaneous tissue and midfoot bones. Red arrow indicates tiny hypointense foci within hyperintense spherical lesions, known as the “dot-in-circle” sign.

The patient was treated with trimethoprim-sulfamethoxazole (TMP-SMX) for eight years, without complete resolution of symptoms, as he had persistent painful nodular draining lesions (Figure 2-A). A new culture was obtained from the draining secretion, and *Gordonia soli* was recovered by culture and identified using 16S rRNA gene sequencing^8^. He was treated with three courses of a 3-week regimen of intravenous linezolid (600 mg every 12 h) plus meropenem (1 g every 8 h) administered at intervals of 24 weeks, and oral TMP-SMX (8 mg/kg/day of the TMP component) was maintained between and after the courses. He experienced significant improvement in pain, edema, and resolution of the purulent discharge.

Following adequate symptom resolution, maintenance therapy with TMP-SMX was discontinued. Four years later, the patient experienced pain recurrence, edema, purulent discharge, and elimination of grains through the fistulas (Figure 2-B). A new specimen was obtained from the secretion, and the grains were observed using direct microscopy (Figure 1-D). The causative organism was cultured (Figure 1-E) and the genus *Gordonia* identified by MALDI-TOF MS (score 1.62) (Bruker MALDI Biotyper), which was confirmed as *Gordonia soli* by 16S rRNA gene sequencing, indicating persistence of infection. Magnetic resonance imaging (MRI) of the foot revealed signs suggestive of inflammation in the subcutaneous tissue; midtarsal and tarsometatarsal joints; and the calcaneum, midfoot, and metatarsal bones, suggesting osteomyelitis. The “dot-in-circle” sign was present, which is suggestive of mycetoma (Figure 2-C). The patient was treated with another two courses of intravenous linezolid plus meropenem, with significant improvement in symptoms, and maintenance therapy was indicated with oral TMP-SMX plus ciprofloxacin (500 mg every 8 h).

## DISCUSSION

In 1971, *Gordona* was identified as a new genus of bacteria from soil and sputum samples using biochemical tests. Initially, it had been classified under the genus *Rhodococcus* due to biochemical similarities. *Gordona* was renamed to *Gordonia* to honor the microbiologist Ruth E. Gordon in 1997^9^.

*Gordonia* is a gram-positive, aerobic, partially acid-fast, fastidious bacterium that requires the use of enrichment media for isolation, and its phenotypic similarities to *Rhodococcus* and *Nocardia* make traditional biochemical identification a challenge^9^. The genus *Gordonia* comprises 39 different species^10^. Similar to other actinomycetes, its slow growth poses a difficulty in microbiological diagnosis, as conventional cultures are typically incubated for only 72 h. There are limitations to the identification of *Gordonia* by MALDI-TOF; therefore, a lower cutoff for identification at the genus (>1.5) and species levels (>1.7) was proposed^10^. Precise identification of genera and species is achieved by molecular methods such as 16S rRNA gene sequencing, which are only available in reference laboratories^6^ ^,^ ^8^ ^,^ ^10^ ^-^ ^11^.

Members of the genus *Gordonia* are present in the soil and can be transferred through aerosols from environmental sources to the respiratory tract, thereby causing pulmonary infections, mainly in immunocompromised hosts. They can also cause bacteremia and spread to the central nervous system, cardiac tissues, and joints. *G. sputi*, *G. bronchialis,* and *G. terrae* are the most frequently identified species in clinical samples^9^.

Actinomycetoma is usually treated with long-term anti-infective medications. Recurrence is common and some patients require combined surgical management to achieve clinical success^1^ ^,^ ^7^. Susceptibility testing for *Gordonia* can be performed using broth microdilutions. Based on isolates reported in literature, it is most predictably susceptible to imipenem, ciprofloxacin, amikacin, linezolid, gatifloxacin, gentamicin and vancomycin^9^. In the present case, susceptibility to TMP-SMX was demonstrated *in vitro* and surgical therapy was considered. However, this was not performed after a shared decision-making approach with the patient, as it was predicted to be mutilating.

Three cases of actinomycetoma caused by *Gordonia* have been reported: two caused by *Gordonia terrae* and one by *Gordonia weatfalia* ^11^ ^,^ ^12^ *.* To the best of our knowledge, this is the first reported case of actinomycetoma caused by *Gordonia soli.*

## References

[1] Zijlstra EE, van de Sande WWJ, Welsh O, Mahgoub ES, Goodfellow M, Fahal AH (2016). Mycetoma: a unique neglected tropical disease. Lancet Infect Dis.

[2] Welsh O, Vera-Cabrera L, Salinas-Carmona MC (2007). Mycetoma. Clin Dermatol.

[3] Emery D, Denning DW (2020). The global distribution of actinomycetoma and eumycetoma. PLoS Negl Trop Dis.

[4] Sampaio FM, Wanke B, Freitas DF, Coelho JM, Galhardo MC, Lyra MR (2017). Review of 21 cases of mycetoma from 1991 to 2014 in Rio de Janeiro, Brazil. PLoS Negl Trop Dis.

[5] Ahmed AA, van de Sande W, Fahal AH (2017). Mycetoma laboratory diagnosis: Review article. PLoS Negl Trop Dis.

[6] Husain U, Verma P, Suvirya S, Priyadarshi K, Gupta P (2023). An overview of mycetoma and its diagnostic dilemma: Time to move on to advanced techniques. Indian J Dermatol Venereol Leprol.

[7] Welsh O, Vera-Cabrera L, Welsh E, Salinas MC (2012). Actinomycetoma and advances in its treatment. Clin Dermatol.

[8] Blaschke AJ, Bender J, Byington CL, Korgenski K, Daly J, Petti CA (2007). Gordonia species: emerging pathogens in pediatric patients that are identified by 16S ribosomal RNA gene sequencing. Clin Infect Dis.

[9] Andalibi F, Fatahi-Bafghi M (2017). Gordonia: isolation and identification in clinical samples and role in biotechnology. Folia Microbiol.

[10] Ercibengoa Arana M, Alonso M, Idigoras P, Vicente D, Marimón JM (2018). Matrix-assisted laser desorption ionization-time of flight mass spectrometry (MALDI-TOF) score algorithm for identification of Gordonia species. AMB Express.

[11] Zampella JG, Kwatra SG, Kazi N, Aguh C (2017). Madura foot caused by Gordonia terrae misdiagnosed as Nocardia. Australas J Dermatol.

[12] Bakker XR, Spauwen PH, Dolmans WM (2004). Mycetoma of the hand caused by Gordona terrae: a case report. J Hand Surg Br.

